# High-Productivity Hybrid Adsorption Desalination Using a Sodium Polyacrylate/CaCl_2_ Composite with Dual Ejectors and Humidification–Dehumidification Under Saudi Arabian Climate Conditions

**DOI:** 10.3390/polym18040450

**Published:** 2026-02-10

**Authors:** Ridha Ben Mansour, Ahmed S. Alsaman, Ehab S. Ali, Ahmed E. Abu El-Maaty, Rached Ben-Mansour

**Affiliations:** 1 Interdisciplinary Research Center for Sustainable Energy Systems, KFUPM, Dhahran 31261, Saudi Arabia; 2 Mechanical Engineering Department, Faculty of Engineering, KFUPM, Dhahran 31261, Saudi Arabia

**Keywords:** sodium polyacrylate, composite, adsorption desalination, polymer–salt adsorbent, humidification–dehumidification (HDH), solar thermal desalination, Riyadh climate, freshwater cost

## Abstract

This study investigates the utilization of a sodium polyacrylate (SP)/CaCl_2_ composite as an adsorbent in a low-grade-heat desalination configuration designed for Saudi Arabian conditions. A dynamic system model was developed and validated for an adsorption desalination (AD) cycle integrated with a dual-ejector and a humidification–dehumidification (HDH) unit. Two operating modes were evaluated, including a production-oriented configuration that applies internal evaporator–condenser heat recovery (HR) when no cooling effect is required. Without HR, the AD–EJ–HDH system achieves 41–56 m^3^/ton·day SDWP and 2.6–2.9 GOR, with a freshwater cost of 1.8–2.4 $/m^3^ under solar driving and 0.70–0.90 $/m^3^ under waste heat. With HR, performance increases to SDWP 95–155 m^3^/ton·day and GOR 2.9–3.1, while costs decrease to about 1.34 $/m^3^ (solar) and 0.38 $/m^3^ (waste heat) in June. The SP/CaCl_2_ composite yields about 85% higher freshwater production than silica gel in the same system, highlighting the material’s potential for high-output hybrid adsorption desalination in hot-climate regions.

## 1. Introduction

Freshwater scarcity has become a binding constraint on socio-economic development in arid and semi-arid regions. In many countries facing water scarcity, particularly in the Gulf region, where large-scale desalination has shifted from being a strategic option to an indispensable pillar of national water security, with seawater desalination now supplying the majority of municipal and industrial freshwater needs [1]. Conventional seawater desalination techniques are subjected to high energy consumption, operating costs, and environmental concerns, in particular, those related to brine discharge [2]. There is a critical need to develop new technologies to address the aforementioned challenges.

Saudi Arabia possesses exceptionally strong solar potential that can support the large-scale deployment of low-grade heat technologies; atlas-based assessments report that the global horizontal irradiation can reach approximately 8.3 kWh/m^2^/day in parts of the Kingdom [3].

These coupled realities motivate the development of new desalination concepts that (i) reduce reliance on electricity-intensive processes, (ii) operate effectively at moderate thermal driving temperatures, (iii) sustain performance under hot ambient conditions, and (iv) minimize the brine discharge.

Thermally driven desalination approaches remain attractive for arid regions when integrated with solar thermal energy or waste heat. Humidification–dehumidification (HDH) is particularly suited to low-temperature heat and small-to-medium scale deployment [4]. Consequently, HDH benefits substantially from hybridization with cycles capable of providing recoverable heat at appropriate temperature levels [5].

Adsorption desalination (AD) has emerged as a leading candidate for such hybridization because it is compatible with low-grade heat sources [6,7] and because its cycle architecture naturally produces a condenser heat stream that can be recovered [8]. However, a persistent limitation of standalone AD is that its freshwater output per unit adsorbent mass can be modest unless advanced recovery and heat-transfer enhancement are applied [9]. Hybrid intensification strategies, most notably ejector enhancement and HDH coupling, have therefore been proposed. Ejector integration has been reported to improve the daily water productivity significantly [10].

Beyond ejector-only intensification, integrated solar-driven configurations combining AD + HDH + two ejectors have been proposed and evaluated, where HDH reuses the adsorption cycle’s waste heat, and ejectors improve the vapor utilization; reported peak outputs reach 98 m^3^/ton·day with GOR about 2.75, while specific water cost can decline to about $1.5/m^3^ in the cited assessment [11].

Another effective method to improve the adsorption performance of the system is the development and the utilization of high-performance adsorbent materials, such as advanced polymers, metal–organic frameworks (MOFs), and covalent organic frameworks (COFs) [12]. In polymer–salt composites, a hydrogel or superabsorbent polymer matrix immobilizes hygroscopic salt solution during hydration, which helps mitigate leakage while maintaining a high working uptake and usable kinetics [13,14]. This concept is directly relevant to CaCl_2_-based sorbents [15,16].

### 1.1. State-of-the-Art Review with Reported Performance Ranges

Recent adsorption–desalination studies have therefore begun to highlight polymeric sorbents, particularly sodium polyacrylate (SP) and SP/CaCl_2_, as promising candidates within solar-temperature ranges; reported benchmarks include SDWP on the order of 45 m^3^/ton·day with COP about 0.67 for SP/CaCl_2_ under solar-driven operating windows [17].

In this context, the adsorbent development becomes a decisive lever. A large fraction of the recent work focuses on salt-based composites (especially CaCl_2_ in porous matrices) to increase the working capacity at moderate regeneration temperatures [18]; yet deliquescence-induced pore blocking and transport penalties remain recurring challenges [19,20].

Advanced adsorbents such as MOFs have demonstrated meaningful productivity gains in modeled and structured-bed concepts; for instance, an MOF-801 system packed in copper foam has been reported to yield 29.7 m^3^/(ton·day) versus 21.5 m^3^/(ton·day) for silica gel under comparable modeled conditions, alongside a higher specific cooling power [21].

The AD system can operate at relatively low heat source temperatures, but the productivity is strongly temperature-dependent. A reported advanced AD cycle produced SDWP about 9.24 m^3^/ton·day at 70 °C, while remaining operable at 50 °C with SDWP 4.3 m^3^/ton·day, and achieving a comparatively high-performance ratio of about 0.77 at the stated condition [22].

Recent solar-driven AD studies reported that SDWP can span wide ranges depending on the configuration and bed design; one synthesis reports typical solar AD SDWP values around 3–5 m^3^/ton·day, while improved heat exchanger/bed configurations can reach SDWP about 23.5 m^3^/ton·day, with SCP about 682 W/kg and COP about 0.32 in a cited high-performance configuration [23,24]. Several studies have introduced the concept of fluidized beds to boost the performance of adsorption-based cooling and desalination systems. For example, Krzywanski et al. [25] proposed a novel approach to tackle the inherent heat and mass transfer limitations by incorporating fluidized bed technology. Their work optimized the thermal and mass transfer behaviors within the adsorbent beds. Another study [26] explored the impact of variables such as the adsorbent particle diameter and fluidizing air velocity. Lasek et al. [27] provided a comprehensive review of a fluidized bed, highlighting its potential to significantly enhance the system performance. In addition, Krzywanski et al. [28] applied automated machine learning (AutoML) techniques to optimize the fluidization parameters in adsorption desalination systems. Furthermore, Krzywanski et al. [29] demonstrated the practical implementation of AI-driven AutoML for diagnostics in existing adsorption systems.

Ejector integration is widely used to intensify vapor utilization, enhance pressure matching, and raise the effective use of low-grade heat. A silica–gel adsorption–ejector combination powered by low-grade heat reported SDWP about 52.67 m^3^/ton at a 95 °C desorption temperature when heat recovery is applied and reported a COP of about 1.47, substantially higher than the non-ejector baseline in that study [30].

HDH is effective at converting low-grade heat into additional distillate, which is why it is frequently hybridized with other cycles for more energy utilization [31]. At the high end of reported integrated concepts, a solar-driven plant combining AD, HDH, and two ejectors reported SDWP about 98.41 m^3^/ton·day with a GOR about 2.75, and the analysis suggested that the specific water cost could reach 0.48 $/m^3^ via waste heat-driven [11]. These performance levels illustrate the potential of full cascade utilization: adsorption produces water directly, ejectors intensify vapor use, and HDH harvests condenser waste heat.

Hybrid AD–HDH systems show strong seasonal peaks in solar-driven assessments, especially when heat recovery is applied and when composite adsorbents are used [32]. A solar-powered ADS integrated with HDH using silica gel/CaCl_2_ reported 69 m^3^/ton·day in June for the heat-recovery hybrid mode, compared to 7.1 m^3^/ton·day for a basic ADS using raw silica gel in the same month.

Advanced adsorbents are increasingly assessed as alternatives to silica gel and salt composites. For MOF-801, a structured-bed optimization study reported a clean-water productivity of about 29.7 m^3^/ton·day compared with about 21.5 m^3^/ton·day for silica gel and reported SCP about 830.8 W/kg compared with about 611.5 W/kg for silica gel under the investigated conditions [21]. For AQSOA/FAM zeolites, the literature base is extensive in terms of adsorption heat transformation and combined desalination/cooling cycles, with multiple studies focusing on the equilibrium and hysteresis behavior that shapes the cycle performance and switching strategy [33,34].

Polymer–salt composites represent a different strategy from rigid porous hosts: the polymer network provides hydrophilicity and structural pathways, while CaCl_2_ supplies strong hydration capacity [35]. In solar ADS-focused reviews, SP/CaCl_2_ is explicitly highlighted as among the most promising materials within solar-regeneration temperature ranges, with a literature-reported SDWP of about 45 m^3^/ton·day, COP about 0.67, and an estimated water cost around $3.8/m^3^ in the cited synthesis [10].

More directly, a recent Saudi-relevant summary of SP-based ADS integrated with HDH reported SDWP about 77.3 m^3^/ton, with water cost values around $1.83/m^3^ for solar-driven operation and $0.49/m^3^ when evaluated with waste heat [36]. These reported values place SP/CaCl_2_ in the performance territory where AD–HDH–ejector cascades can plausibly benefit from higher vapor throughput. Accordingly, the present work is positioned to advance a climate-driven hybrid desalination concept tailored for high solar irradiation regions, which combines the following: (i) SP/CaCl_2_ composite adsorption beds, (ii) ejector-based vapor utilization enhancement, and (iii) HDH recovery of condenser waste heat. The underlying objective is to translate the material-level adsorption advantage into system-level gains in annual productivity, GOR, and cost under Saudi Arabian climatic conditions, where the high solar potential can support adsorbent regeneration.

### 1.2. Novelty and Contributions

In response to the above gaps, SP/CaCl_2_ has been assessed mainly in conventional and heat-recovery AD configurations, while ejector–HDH hybridization has been demonstrated primarily with silica gel beds. This paper presents an integrated framework for intensifying desalination under Saudi Arabian climate conditions. The main novelties and contributions can be summarized as follows:

– An SP/CaCl_2_-based adsorption desalination cycle integrated with two ejectors (V–V and L–V) and an HDH loop, building on the validated AD2EJ–HDH architecture, previously demonstrated with silica gel, and on the experimentally supported superior uptake/kinetic behavior of SP/CaCl_2._
– Saudi-climate-driven assessment: implementation of a year-round performance analysis under representative Saudi weather, explicitly capturing how high ambient temperature and seasonal irradiation influence the adsorption equilibrium, condenser heat availability to the HDH unit, and ejector-driven vapor utilization (a step not addressed in the cited Egypt-based hybrid demonstrations).
– Thermo-economic model for tri-hybrid operation: formulation of a system-level model that links SP/CaCl_2_ dynamic adsorption behavior (including fitted kinetic parameters) with ejector and HDH sub-models as adopted in AD2EJ–HDH feasibility studies, enabling the consistent prediction of SDWP, GOR, and specific water cost.
– Identification of the optimal operating parameters (cycle time, regeneration temperature, and HR arrangement) for the AD2EJ–HDH literature utilizing SP/CaCl_2_.
– Benchmarking against state-of-the-art adsorbents and hybrids: quantitative comparison against silica gel-based AD2EJ–HDH performance levels and against salt-composite sorbents reported for adsorption desalination/cooling applications, clarifying when SP/CaCl2 offers a genuine advantage for ejector–HDH intensification.

## 2. Materials and Methods

### 2.1. Composite Material Preparation

Sodium polyacrylate (SP) was selected as the baseline adsorbent. SP is the sodium salt of polyacrylic acid and behaves as an anionic polyelectrolyte with negatively charged carboxylic groups along the polymer backbone. Owing to the presence of sodium ions, SP can absorb large quantities of water (reported up to about 300 times its mass), while maintaining favorable mechanical stability, strong hydration capability, and high heat resistance; moreover, it is considered non-toxic. The SP used in this work was supplied as white granules with a particle size range of 0.075–0.8 mm (approximately 74% between 0.3 and 0.6 mm) and a bulk density of 720–760 kg·m^−3^ [17].

**Figure 1:**
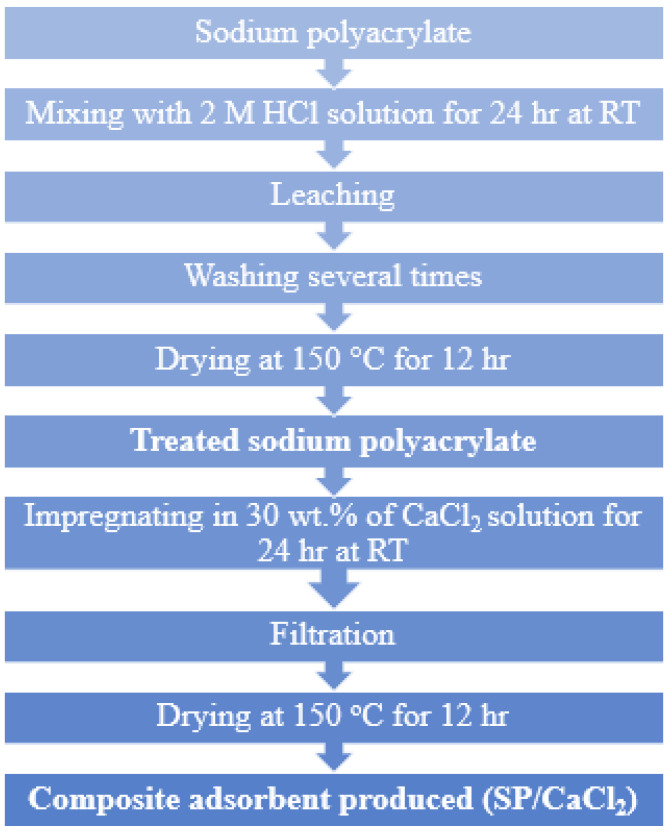
SP/CaCl_2_ adsorbent material preparation method [17].

To modify the adsorption characteristics, the SP-based materials were prepared using hydrochloric acid (HCl) and calcium chloride (CaCl_2_). Acid treatment employed 2 M HCl (selected as an optimum concentration in the referenced procedure). For salt-based activation/composite preparation, dried SP (2.1 g) was impregnated with a 30 wt% loading of CaCl_2_, previously dissolved in distilled water, using a direct impregnation approach [17]. The preparation method is illustrated in Figure 1, while detailed information on the material synthesis and adsorption characterization is listed in the Supplementary File.

### 2.2. System Description

**Figure 2:**
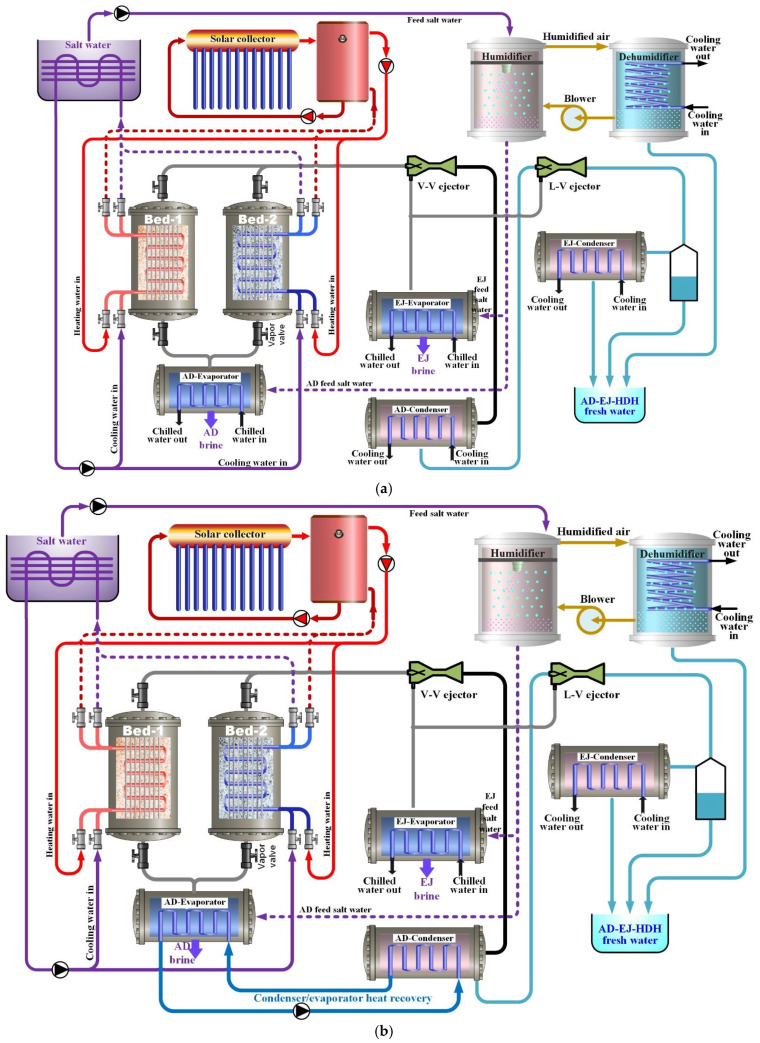
Proposed SP/CaCl_2_ AD-EJ-HDH hybrid system: (**a**) without evaporator condenser heat recovery (with additional cooling effect); (**b**) with evaporator condenser heat recovery (without cooling effect).

The proposed system is a tri-hybrid desalination system (SP/CaCl_2_ AD-EJ-HDH) that couples adsorption desalination (AD) with two ejectors and a humidification–dehumidification (HDH) unit. As illustrated in Figure 2, two different configurations (1 and 2) were designed to maximize the freshwater production and reduce the amount of rejected brine by the cascading utilization of low-grade heat. Solar thermal energy supplies the regeneration heat required for the desorption, while the condenser waste heat and outlet cooling water released during the adsorption phase, typically rejected to the environment, are recovered and harvested to drive the HDH process.

In configuration 1, illustrated in Figure 2a, a solar collector field heats water that serves as a heat-transfer fluid (HTF). The heated HTF flows through a thermal storage or buffer tank before entering a heat exchanger. This buffer reduces the short-term fluctuations in solar input and stabilizes the desorption temperature delivered to the adsorption beds.

Seawater is introduced to a low-pressure evaporator (AD-evaporator) and a relatively high-pressure evaporator (EJ-evaporator), where it evaporates at a reduced temperature under vacuum conditions in both units. The generated vapor stream is directly adsorbed by the active bed from the AD-evaporator (≈1 kPa) or routed to the ejector suction side from the EJ-evaporator (≈2.25 kPa). Concentrated brine exits the evaporator and is managed through controlled discharge or routing into the HDH loop.

In general, the adsorption system core consists of two adsorption beds (I and II) packed with SP/CaCl_2_ composite within a finned-tube heat exchanger. The beds operate in an alternating manner: during operation, one bed undergoes adsorption while connected to the AD evaporator, while the other receives hot HTF to drive the desorption phase. This alternating operation ensures quasi-continuous freshwater production.

Two ejectors are installed downstream of the adsorbent bed vapor lines to enhance the vapor utilization through pressure recovery and the entrainment of low-pressure vapor: the first is a vapor–vapor ejector (V-V ejector), in which a high-pressure vapor motive released during the desorption phase entrains low-pressure vapor generated in the EJ-evaporator and discharges the mixed vapor at a higher intermediate pressure. The second is a liquid–vapor ejector (L-V ejector), which further enhances the entrainment and discharge pressure matching by employing a suitable liquid motive stream produced in the AD condenser. The L-V ejector is arranged between the liquid condenser outlet (primary nozzle) and the EJ evaporator line (secondary nozzle), as illustrated in Figure 2. Together, the ejectors increase the effective use of the available vapor, reduce throttling losses, and improve the thermodynamic integration between the desorbed vapor generator and the condenser, especially under low-grade heat operating conditions.

In the condenser, the discharge stream released from the L–V ejector is condensed to produce high-quality distillate. A key design feature of the proposed configuration is that the condenser and cooling water discharged during the adsorption phase are treated as a recoverable heat source rather than a thermal sink. The waste heat is then transferred to the HDH feed stream, increasing the thermal driving potential of the HDH process. As a result, both the external solar heating demand and the external cooling requirement are reduced, leading to more effective utilization of solar heat.

The HDH unit typically contains a packed humidifier and a dehumidifier. In the humidifier, preheated saline water comes into contact with an airstream, increasing the moisture content of the air. The humidified air then passes to the dehumidifier, where cooling induces condensation and produces additional desalinated water. A controlled blowdown stream is employed to limit the salinity buildup in the loop and maintain stable system operation.

Figure 2b presents Configuration 2, which is intended for operating periods when cooling is not required, and the main objective is to maximize the freshwater production. In this mode, an internal evaporator–condenser heat recovery (HR) pathway is activated within the adsorption desalination loop, allowing the latent heat released during vapor condensation to be recovered rather than ejected into the environment. This recovered heat is transferred to the AD evaporator to support additional evaporation. Such heat cascading increases the effective utilization of the supplied low-grade thermal energy, strengthens the evaporation–adsorption driving potential, and enlarges the useful uptake swing of the SP/CaCl_2_ beds during cyclic operation. As a result, Configuration 2 prioritizes water production by converting internally available thermal energy into additional vapor generation, thereby improving the overall desalination productivity under the same external heat input conditions.

### 2.3. Theoretical Model

#### 2.3.1. Assumptions

To solve the governing equations with a reasonable level of model simplicity while maintaining solution accuracy, a set of standard assumptions was adopted [37].

- The desalination plant is considered well insulated; so, heat losses to the surroundings are neglected.
- The specific heat capacities of the working materials are assumed to be constant over time.
- The condenser outlet is treated as saturated liquid, whereas the evaporator outlet is assumed to be saturated vapor.
- Flow through the ejectors is modeled as one-dimensional, and ejector irreversibilities, such as frictional effects and non-ideal mixing, are accounted for using isentropic efficiency definitions.
- For the HDH subsystem, the outlet air from both the humidifier and dehumidifier is assumed to be saturated.

#### 2.3.2. Governing Equations

The adsorption–ejector subsystem employed in this study follows the modeling framework reported in previous work [11]. Accordingly, the same transient formulation is used, based on coupled energy balance equations and water-vapor uptake kinetics. The main governing relations are summarized in Table 1.

For the humidifier and dehumidifier subsystem, the governing equations are summarized in Table 2, where each component is modeled using its effectiveness-based formulation [38].

The ejector is modeled by applying the conservation of mass, momentum, and energy across its main sections, namely the primary nozzle, suction chamber, mixing zone, and diffuser. Accordingly, the governing relations for the flow through the primary nozzle are expressed as follows [40]:

(20) $$P_{p^{\prime }}=P_{p}-\Delta P_{pn}$$

(21) $$h_{p^{\prime },i}=h(P_{p^{\prime }},s_{p})$$

(22) $$h_{p^{\prime }}=h_{p}+\eta_{pn}(h_{p^{\prime },i}-h_{p})$$

(23) $$\nu_{p^{\prime }}=\sqrt{2(h_{p}-h_{p^{\prime }})}$$

where $P_{p^{\prime }}$ is the pressure at the exit, $P_{p}$ is the pressure at the inlet, and $\nu_{p\prime }$ is the exit velocity. $s_{p}$ is the specific entropy at the inlet, $\eta_{pn}$ is the nozzle efficiency, and $h_{p}$ is the specific enthalpy at the inlet.

Similarly, the equations of the secondary nozzle are written as follows:

(24) $$P_{s^{\prime }}=P_{s}-\Delta P_{sc}$$

(25) $$h_{s^{\prime },i}=h(P_{s^{\prime }},s_{s})$$

(26) $$h_{s^{\prime }}=h_{s}+\eta_{sc}(h_{s^{\prime },i}-h_{s})$$

(27) $$\nu_{s^{\prime }}=\sqrt{2\left(h_{s}-h_{s^{\prime }}\right)}$$

(28) $$P_{s\prime }=P_{p\prime }=P_{m}$$

where $P_{m}$ is the pressure in the mixing section, and the velocity and enthalpy of the fluid in the mixing section can be estimated as

(29) $$\nu_{m}=\eta_{m}\frac{\nu_{s\prime }+ER\cdot \nu_{s\prime }}{1+ER}$$

(30) $$\;h_{m}=\frac{h_{p}+ER\cdot h_{s}}{1+ER}$$

(31) $$s_{m}=s(P_{m},h_{m})$$

The pressure and enthalpy across the normal shock can be given as

(32) $$P_{as}+\rho_{as}\nu_{as}^{2}=P_{m}+\rho_{m}\nu_{m}^{2}$$

(33) $$h_{as}+\frac{1}{2}\nu_{as}^{2}=h_{m}+\frac{1}{2}\nu_{m}^{2}$$

(34) $$s_{as}=s(P_{as},h_{as})$$

(35) $$h_{e}=h_{m}+0.5\;\eta_{d}\;\nu_{m}^{2}$$

(36) $$P_{e}=P(h_{e},s_{m})$$

where v denotes the flow velocity, and ER is the ejector entrainment ratio. The subscripts p, s, and m correspond to the primary, secondary, and mixed streams, respectively. The subscript as indicates the state immediately downstream of the normal shock, while e refers to the ejector outlet conditions.

In accordance with the second law of thermodynamics, the entropy generation within each component, as well as for the overall system, must be non-negative for all operating conditions. Therefore, an entropy-generation relation is evaluated in this study to confirm that the predicted results are thermodynamically consistent and comply with the fundamental constraints imposed by the second law.

(37) $${\dot{S}}_{gen,k}=\sum_{out,k}\left(\dot{m}s\right)-\sum_{in,k}\left(\dot{m}s\right)\geq 0$$

To assess the economic viability of the hybrid desalination system, a cost model of the desalinated water was developed. Equations (38)–(42) summarize the main relations used to perform the economic analysis, while Table 3 presents the capital cost of the individual components of the proposed system:

(38) $${\dot{Z}}_{T}={\dot{Z}}_{c}+{\dot{Z}}_{l}+{\dot{Z}}_{m/o}\;\;\;\left(in\;\frac{\$}{yr}\right)$$

(39) $${\dot{Z}}_{c}=Z_{c}\times F\;\left(in\;\frac{\$}{yr}\right)$$

(40) $$F=\frac{r{\left(r+1\right)}^{n}}{{\left(r+1\right)}^{n}-1}\;\left(in\;\frac{1}{yr}\right)$$

(41) $${\dot{Z}}_{l}=L\times {\dot{Z}}_{c}\;\left(in\;\frac{\$}{yr}\right)$$

(42) $${\dot{Z}}_{m/o}=M\times {\dot{Z}}_{c}\;\left(in\;\frac{\$}{yr}\right)$$

where n is the number of operation years (30 y), r is the interest rate (5%), F is the amortization charge, L is the labor cost percentage (8.11%), and M is the maintenance cost percentage (4.63%) [41]. The power of the blowers and pumps is 1.38 kWh/m^3^ [41].

The model validation and simulation procedure are attached in the Supplementary File.

## 3. Results

**Figure 3:**
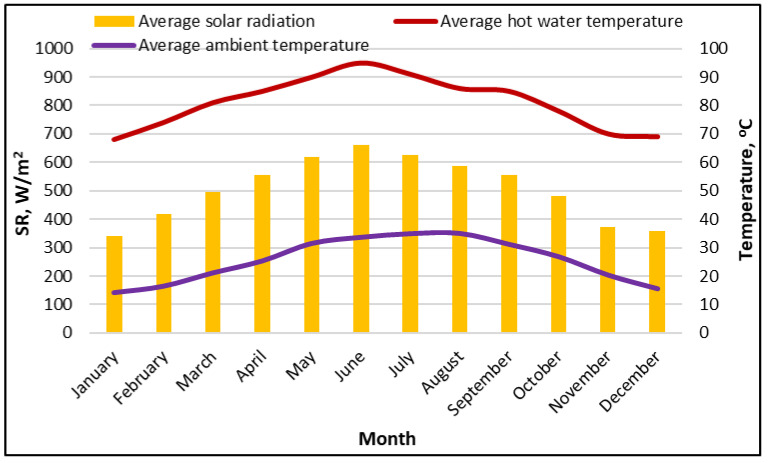
Daily solar radiation, ambient temperature, and average hot water temperature for Riyadh city extracted from TRNSYS software.

This section presents the results of utilizing SP-CaCl_2_ in hybrid AD-EJ-HDH in Saudi Arabian weather conditions. Figure 3 shows a clear seasonal pattern that is typical of Riyadh’s arid climate. The average daily solar radiation increases steadily from winter to early summer, rising from about 340W/m^2^ in January to a peak of roughly 660 W/m^2^ in June. After mid-summer, the solar radiation decreases gradually, falling to around 360 W/m^2^ by December. The figure also shows the average daily ambient temperature of Riyadh city extracted from TRNSYS software.

The hot-water temperature (representing the solar-heated driving source delivered to the system) tracks the solar availability. It increases from roughly 68 °C in January to a maximum of about 95 °C in June and then declines to 69 °C by December. This profile is important for system operation, because it determines the feasible regeneration (desorption) temperature window and the stability of continuous operation. The summer months provide the strongest thermal driving potential for adsorption regeneration. This seasonal coupling explains why a hybrid configuration that reuses condenser heat to drive the HDH and applies heat recovery between beds (configuration 2) becomes especially valuable in hot climates, where the thermal gradients for cooling are reduced.

**Figure 4:**
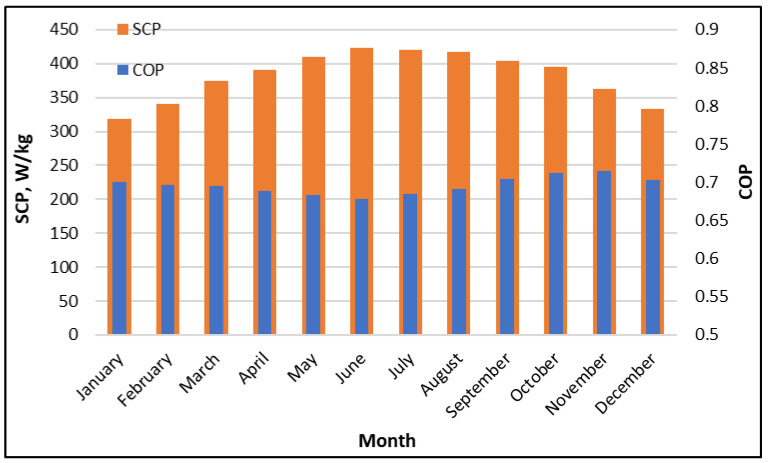
Monthly AD-system SCP and COP throughout the year at Riyadh, utilizing SP/CaCl_2_.

Figure 4 expresses the monthly trends of the specific cooling power (SCP) and coefficient of performance (COP) of the proposed hybrid system. The figure shows the response of the adsorption subsystem to the seasonal variation in driving and ambient conditions. The SCP increases from winter to summer, rising from about 320 W/kg in January to about 420 W/kg during May-September period, with peak values occurring when the available hot-water temperature is also the highest. This behavior is expected, as stronger regeneration temperature improves the desorption process, increases the working uptake swing, and raises the flow rate of vapor absorbed or desorbed during the sorption phase. The SCP then declines, reaching approximately 330 W/kg in December, consistent with reduced solar input and lower driving temperature in the winter season.

In contrast, the coefficient of performance (COP) varies within a narrower band and shows less seasonal sensitivity. Throughout the year, the COP values remain approximately between 0.66 and 0.72, with a slight minimum observed from the late spring to early summer (0.66) and higher values during the cooler months, reaching about 0.7- 0.72 in the October–December period. This pattern of COP reflects a balance between the useful effect and thermal energy input. While higher driving temperatures enhance the cooling capacity (as SCP), they also increase the thermal energy supplied to the beds, while high ambient conditions reduce the adsorption-side cooling effectiveness and increase irreversibilities. As a result, the system delivers higher cooling capacity in summer, but the efficiency gains are partially offset by larger heat input and more demanding heat rejection conditions. This behavior underscores the engineering rationale for implementing bed-to-bed heat recovery and condenser heat reuse within the HDH subsystem in the full hybrid configuration.

**Figure 5:**
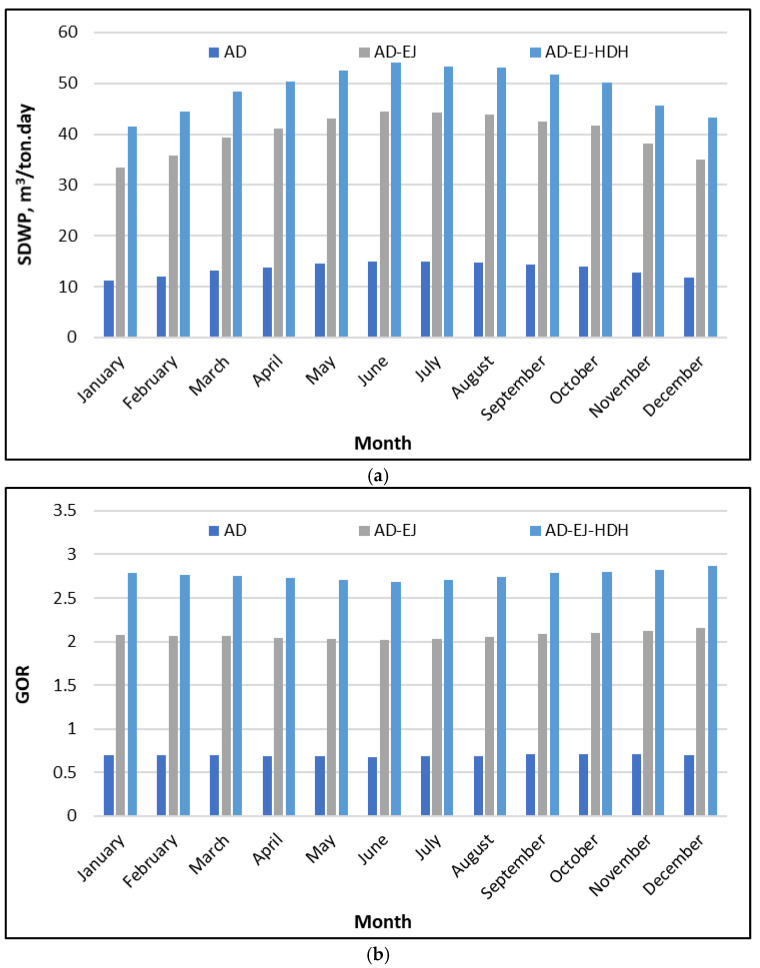
GOR of the proposed SP/CaCl_2_ AD-EJ-HDH system compared to AD and AD-EJ throughout the year at Riyadh: (**a**) SDWP; (**b**) GOR.

Figure 5a illustrates that the SDWP profiles show a clear and consistent ranking across the whole year. The baseline AD system remains in the range of roughly 11–15 m^3^/ton·day, with lower values in winter (11–12 m^3^/ton·day in January–February) and a modest rise toward late spring and summer (14–15 m^3^/ton·day in May–September). Adding the ejector stage (AD–Ejector) shifts the productivity to a much higher band, typically around 33–45 m^3^/ton·day. The highest monthly values appear in the warm season, reaching about 44–45 m^3^/ton·day around June–September, while the winter months drop to about 33–36 m^3^/ton·day. The full tri-hybrid configuration (AD–EJ–HDH) provides the largest output throughout the year, rising from approximately 41–45 m^3^/ton·day in winter to about 52–55 m^3^/ton·day in the peak months, with the maximum occurring around June–September.

These results indicate that the productivity gains are not incremental; they are structural. Relative to AD alone, the ejector-assisted case increases the SDWP by about a factor of 300% (e.g., 15 to 45 m^3^/ton·day in summer). This reflects stronger vapor handling and improved utilization of the available driving heat in the AD loop. The additional step from AD–EJ to AD–EJ–HDH typically adds another 10–15 m^3^/ton·day, which corresponds to a roughly 25% uplift depending on the month (e.g., 45 to 55 m^3^/ton·day). This extra water is best explained by recovering the condenser heat and converting it into useful evaporation capacity inside the HDH unit, rather than rejecting it to the environment. The seasonal pattern further supports this interpretation, as the highest SDWP coincides with hot months, where the available solar source is highest, and the system can sustain stronger desorption and more effective circulation of thermal energy.

Figure 5b compares the monthly GOR for the AD, AD–EJ, and AD–EJ–HDH configurations. The obtained GOR trends are consistent with the productivity ranking while providing additional insight into the thermal energy utilization. The baseline AD system exhibits a relatively low GOR, remaining in the range of approximately 0.65–0.72 throughout the year. The integration of ejectors (AD–EJ) increases the GOR to a stable band near 2.0–2.15, with only minor seasonal changes. The full hybrid configuration (AD–EJ–HDH) achieves the highest GOR values, generally between 2.6 and 2.9. The peak values occur toward late summer and early winter, reaching approximately 2.75–2.85, while the minimum values, around 2.6–2.65, are obtained in early summer.

From an energy perspective, these results confirm that the hybrid design improves not only water output but also the effectiveness of thermal energy utilization. Transition from the base AD configuration to AD–EJ increases the GOR by roughly a factor of three, indicating that ejector integration and improved vapor management substantially reduce heat losses and increase the useful latent output per unit heat supplied. Further integration of the HDH unit (AD–EJ–HDH) raises the GOR by an additional 0.6–0.8, which corresponds to an improvement of about 30–40% during many months. This gain is consistent with the role HDH unit acting as a heat upgrader, where the condenser heat is recovered and used to drive extra humidification and condensation. The relatively smooth month-to-month variation in the GOR also suggests that the system’s efficiency is not overly sensitive to seasonal conditions, even though the absolute productivity (SDWP) increases during periods with higher driving temperature and higher solar availability.

**Figure 6:**
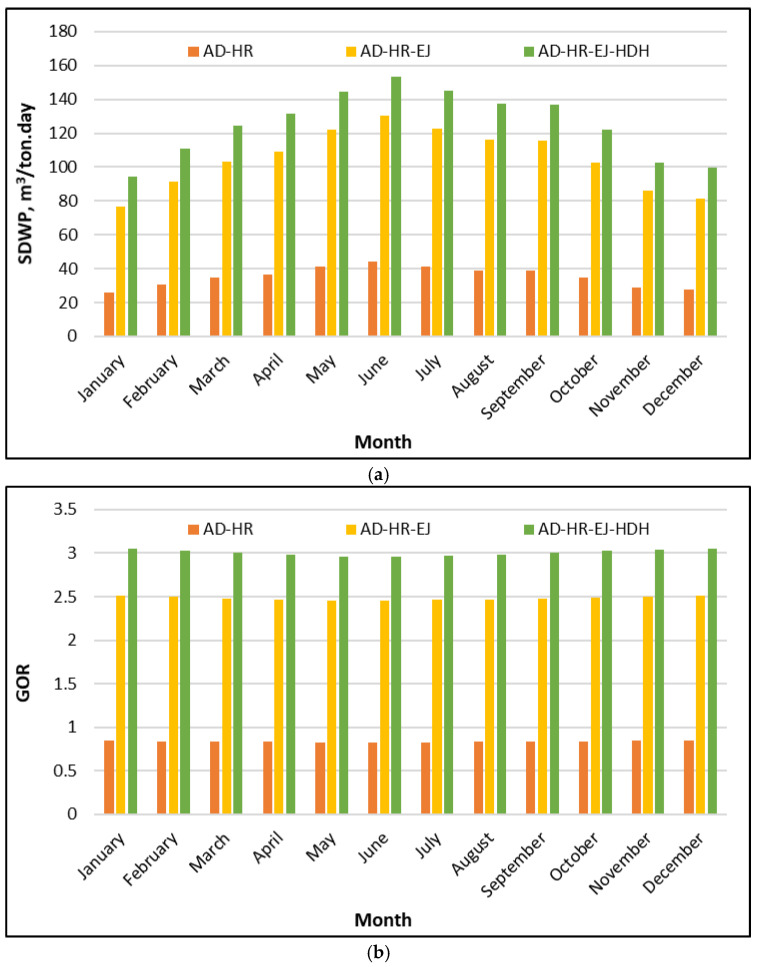
GOR of the proposed SP/CaCl_2_ AD-HR-EJ-HDH system compared to AD-HR and AD-HR-EJ throughout the year in Riyadh: (**a**) SDWP; (**b**) GOR.

In Configuration 2, an evaporator–condenser heat-recovery (HR) scheme is implemented to maximize the freshwater production. This operating mode is intended for periods when the cooling demand is absent, such that the recovered heat is redirected to sustain evaporation and enhance the desalination yield rather than to produce a cooling effect. Figure 6a shows that applying heat recovery (HR) inside the adsorption cycle creates a stable baseline SDWP, and adding ejectors and HDH produces a significant improvement. The AD-HR case remains the lowest, ranging approximately from 25 to 44 m^3^/ton·day throughout the year. The minimum appears in winter (about 25–30 m^3^/ton·day in January–February and December), while the maximum occurs in early summer at about 43–45 m^3^/ton·day in the May–June period. When ejectors are integrated to form the hybrid system AD-HR-EJ, the SDWP rises substantially into the range of 80–130 m^3^/ton·day. The highest values occur during the May–June period with an SDWP of 120–130 m^3^/ton·day, whereas the winter values fall to about 80–90 m^3^/ton·day. With the full configuration (AD-HR-EJ-HDH), SDWP attains the highest water production throughout the year, ranging from 95 to 155 m^3^/ton·day. A pronounced peak is obtained around June, reaching 155 m^3^/ton·day), whereas lower, but still high, values of approximately 95–103 m^3^/ton·day occur during January and December.

From an engineering standpoint, the improvement pattern is physically consistent in that each integration stage strengthens the heat and vapor cascade. Integration of the HR reduces the external heat input required for regeneration by reusing internal thermal energy from the condenser–evaporator/adsorption circuit. As a result, the SDWP is directly increased compared with the non-recovery operation. Further integration of the ejector stage improves the water productivity by enhancing the vapor handling and pressure matching, allowing a large fraction of the generated vapor to be converted into useful condensation rather than being limited by throttling or pressure losses. The final increase when transitioning from AD-HR-EJ to AD-HR-EJ-HDH reflects the contribution of HDH, which converts the condenser heat that would otherwise be rejected into additional freshwater. The effect is more pronounced during warmer months, when higher driving temperatures and enhanced vapor generation potential enable more effective cascading utilization of low-grade thermal energy.

Using the SP/CaCl_2_ composite adsorbent, the proposed system achieves a freshwater productivity approximately 85% higher than that obtained with silica gel under identical configuration and operating conditions. This substantial gain highlights the strong potential of SP/CaCl_2_ to enhance the hybrid adsorption desalination performance and supports the relevance of the present study for high-output operation in Saudi climatic conditions.

Figure 6b compares the thermal utilization efficiency using GOR, and it confirms the same ranking seen in the SDWP. The AD-HR configuration shows GOR values around 0.8–0.9, with only small month-to-month changes. When ejectors are integrated (AD-HR-EJ), the GOR rises sharply to approximately 2.4–2.5, again with limited seasonal fluctuation. The full hybrid (AD-HR-EJ-HDH) achieves the highest GOR, clustered close to 2.9–3.1 over the year, and it remains consistently above the other configurations. The key point is that the efficiency improvement is not limited to summer; the GOR enhancement persists across all months, indicating that the hybridization improves the energy utilization and not only the peak-month output.

This GOR behavior can be explained by how the three configurations manage heat recovery and reuse. In the AD-HR configuration, the recovered heat reduces the net thermal input required per unit of distillate, which improves the GOR even though the absolute water production remains moderate. In the AD-HR-EJ configuration, ejector integration enhances the vapor utilization efficiency, allowing a larger fraction of the supplied thermal energy to be converted into the latent heat of condensed freshwater rather than being lost through thermodynamic irreversibility. In the full AD-HR-EJ-HDH configuration, the condenser heat is upgraded into additional evaporation/condensation work inside the HDH loop, which increases the useful output without a proportional increase in external heat input. This produces the highest GOR and explains why the curve remains high and stable through the year. Practically, this result is important for Riyadh operations, because it suggests that the proposed tri-hybrid system can maintain strong thermal efficiency even when seasonal conditions shift, while still capturing peak productivity in high-irradiance months.

**Figure 7:**
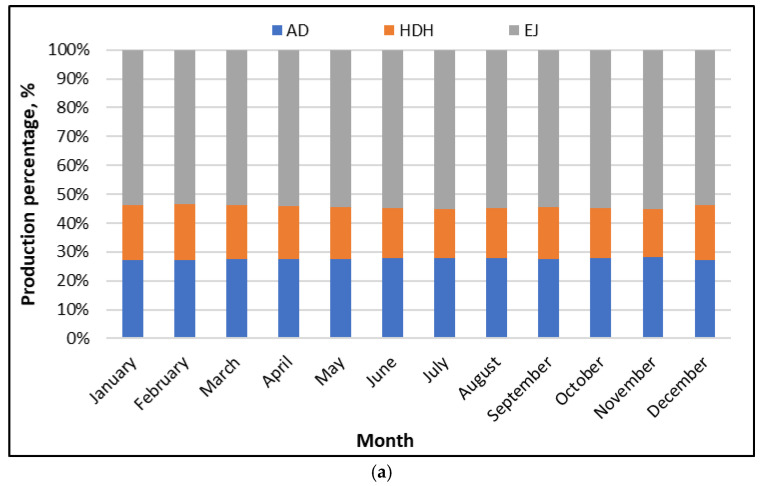

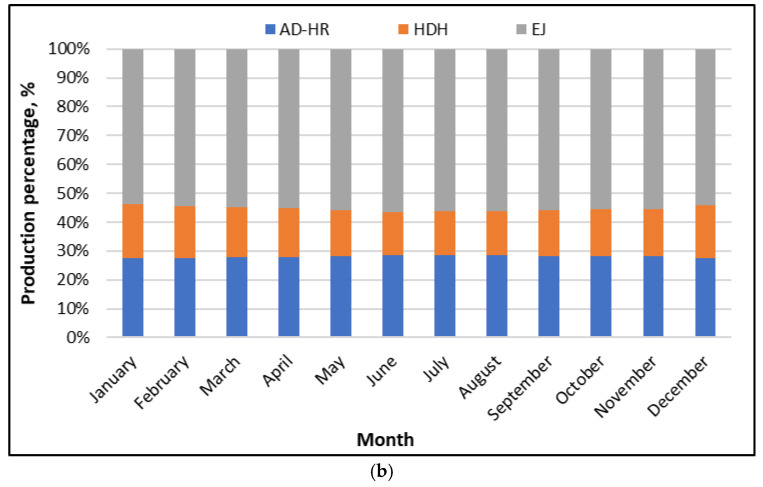
Subsystem’s production shares of SP/CaCl_2_ AD-EJ-HDH system throughout the year at Riyadh: (**a**) AD-EJ-HDH; (**b**) AD-HR-EJ-HDH.

Figure 7a presents the average of the subsystems’ freshwater production share for the SP/CaCl2 AD-HR-EJ-HDH system compared to AD-HR and AD-HR-EJ throughout the year at Riyadh. The distribution is remarkably stable over the year. The AD share remains close to 26–28%, with only minor month-to-month variation. The HDH share typically falls in the range of 17–20%. The EJ share is consistently the largest component, accounting for about 52–56% of total production. This means that, in every month, more than half of the delivered freshwater is linked to the ejector-assisted intensification pathway.

The stability of these fractions is an important engineering observation. It indicates that the three subsystems scale in a coordinated way, as the driving conditions change seasonally. The ejector-related share dominates, because the ejector train improves vapor utilization and pressure matching, which increases the amount of vapor that can be effectively condensed into product water. Meanwhile, HDH provides a meaningful but smaller fraction, because its output is constrained by air–water contact effectiveness and by the available temperature approach in the dehumidifier. The AD block maintains a steady baseline contribution, because it is primarily governed by the adsorbent working capacity and switching strategy. Overall, the figure shows that the hybrid system does not rely on a single component; instead, it distributes production across parallel mechanisms, with the ejector providing the strongest intensification effect.

Figure 7b shows the same production-share analysis when internal heat recovery (HR) is added within the AD loop. Here, the AD-HR share stays around 27–30%, which is slightly higher than the AD share in Figure 7a. The HDH share remains close to 15–18%, which is marginally lower than the non-HR case. The EJ share continues to dominate at roughly 53–58%, remaining the largest fraction in every month. As in the non-HR configuration, seasonal fluctuations are small, and the shares remain well clustered throughout the year.

This shift in shares has a clear physical meaning. When HR is applied inside the AD system, more of the internally available heat is reused to support evaporation and regeneration within the adsorption cycle. That tends to strengthen the AD contribution and reduce the amount of “excess” recoverable heat that would otherwise be routed to HDH; so, the HDH fraction can decrease slightly, even if the absolute HDH production remains high. The ejector contribution stays dominant, because its role is tied to vapor handling and condensation effectiveness, which remains central in both configurations. In practice, Figure 7b suggests that HR strengthens the AD core without destabilizing the overall production balance, which is desirable for Riyadh operation, where the seasonal temperature swings can challenge heat rejection and cycle control.

**Figure 8:**
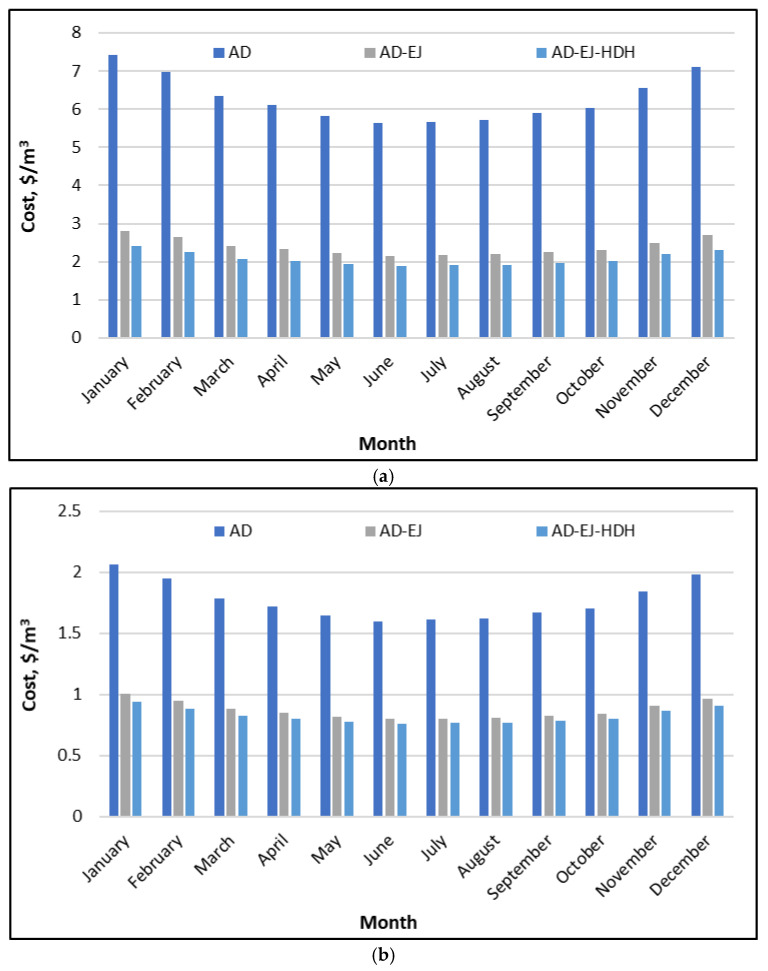
Freshwater cost of the proposed SP/CaCl_2_ AD-EJ-HDH system compared to AD and AD-EJ throughout the year at Riyadh: (**a**) powered by solar energy; (**b**) powered by waste heat.

Figure 8a compares the unit freshwater cost of three configurations: AD, AD–EJ, and AD–EJ–HDH—when solar energy is used to supply the regeneration heat, and no internal heat recovery is applied within the AD loop. The baseline AD configuration shows the highest costs throughout the year, varying from about 5.6 $/m^3^ in June, up to roughly 7.4 $/m^3^ in winter, particularly January. This seasonal pattern follows the availability of solar resources and the resulting productivity: higher solar radiation and hot-water temperature increase the water productivity, for the same capital investment, thereby reducing the unit cost. Incorporation of an ejector (AD–EJ) leads to a substantial reduction in the unit water cost, reducing it to about 2.0–2.6 $/m^3^, with the minimum values occurring in the high-irradiance months (approximately 2.0–2.2 $/m^3^ between May and September) and higher values in winter (about 2.4–2.6 $/m^3^). The full hybrid (AD–EJ–HDH) achieves the lowest costs, typically around 1.8–2.4 $/m^3^, with the summer months close to 1.8–2.0 $/m^3^ and the winter values rising toward 2.3–2.4 $/m^3^.

In general, the unit cost of fresh water produced by a solar-driven desalination system is strongly governed by the ratio of the annual water production to the combined capital and operating expenses of solar collectors, thermal storage, and desalination components. The integration of ejectors and the HDH unit reduces the unit cost primarily by increasing the specific daily water production (SDWP) without a proportional increase in the solar collection area. The ejector improves vapor utilization and effective condensation, while the HDH unit converts waste condenser heat into additional distillate water. Consequently, the hybrid configuration maintains a consistent cost advantage throughout the year. The figure also indicates that the hybrid architecture partially mitigates seasonal cost fluctuation. Although winter costs remain higher, the difference between winter and summer costs is significantly smaller for the AD–EJ and AD–EJ–HDH configurations than for the baseline AD system, reflecting more efficient utilization of the available solar energy.

Figure 8b shows the same considered configurations when the system is driven by waste heat, again assuming no internal heat recovery. Under these conditions, the water costs are much lower than in the solar-driven case, as the thermal energy input is considered low-cost or essentially free, leaving capital recovery and auxiliary electricity as the dominant cost components. The AD configuration exhibits a unit cost of about 1.6–2.0 $/m^3^. The AD–EJ configuration further reduces the cost to approximately 0.75–0.95 $/m^3^, while the fully integrated AD–EJ–HDH system becomes the least expensive option, typically in the range of 0.70–0.90 $/m^3^. In summer, costs approach 0.70–0.75 $/m^3^ with a modest increase toward winter.

Although the relative ranking of configurations and the general seasonal trends remain consistent with the solar-driven case, the underlying cost drivers differ. When waste heat is used, sensitivity to solar resource availability is eliminated, and the remaining seasonal variation is primarily governed by ambient conditions that affect condensation temperature levels, bed cooling effectiveness, and overall cycle productivity. *The* continued economic advantage of ejector and HDH integration reflects their ability to increase freshwater output. Importantly, the gap between AD–EJ and AD–EJ–HDH remains meaningful, indicating that the HDH contribution is not marginal; it systematically converts available condenser heat into additional freshwater even when the primary heat input is not costly. In practice, these results indicate that the proposed hybrid system is especially attractive for Saudi industrial sites where waste heat is available, offering both high productivity and low unit water cost under realistic operating conditions.

**Figure 9:**
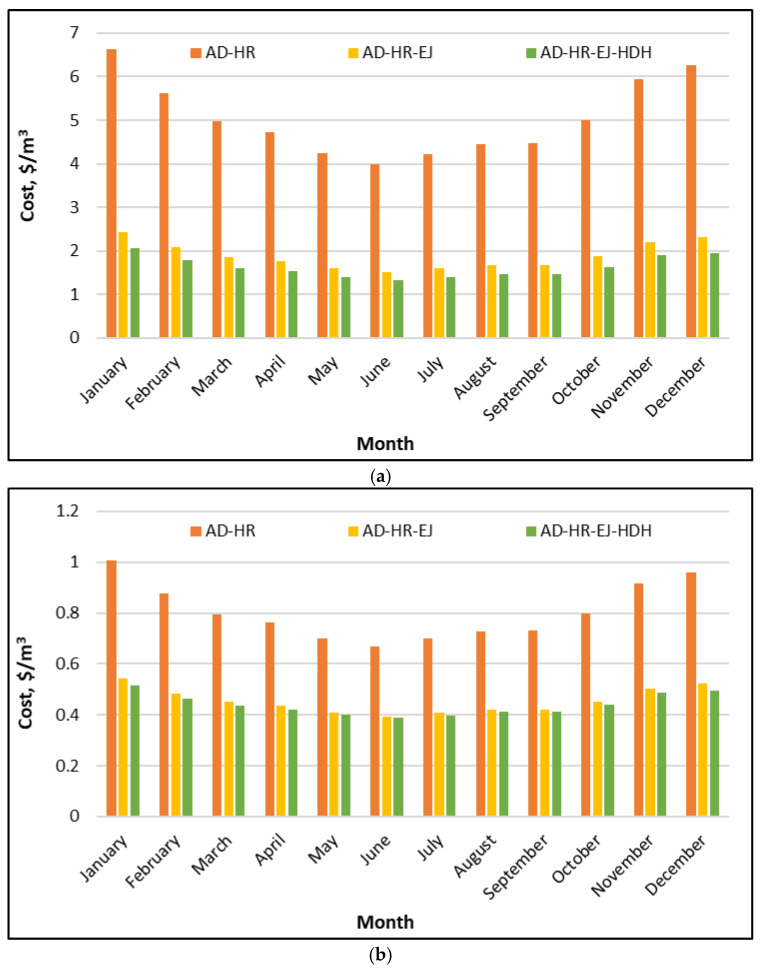
Freshwater cost of the proposed SP/CaCl_2_ AD-HR-EJ-HDH system compared to AD-HR and AD-HR-EJ throughout the year in Riyadh: (**a**) powered by solar energy; (**b**) powered by waste heat.

Figure 9a shows that introducing internal heat recovery (HR) within the adsorption–evaporator–condenser loop reduces the unit freshwater cost for all configurations, while preserving the same ranking among them. The AD-HR case remains the most expensive, varying from about 4.0 $/m^3^ in June (the minimum) to roughly 6.6 $/m^3^ in January (the maximum). A clear seasonal trend appears: costs decrease from winter into late spring and summer and then rise again toward autumn and winter. When the ejector is added (AD-HR-EJ), the cost drops to a narrower band of approximately 1.51–2.4 $/m^3^, with minimum values around 1.51 $/m^3^ in June and higher values near 2.3–2.4 $/m^3^ in January and December. The full tri-hybrid (AD-HR-EJ-HDH) gives the lowest cost in every month, typically around 1.33–2.1 $/m^3^, with its best months close to 1.3–1.4 $/m^3^ in the high-irradiance season and winter values around 1.9–2.1 $/m^3^.

The engineering interpretation is that HR reduces the net external heat requirement per cycle, which increases the water production per unit solar heat collected and lowers the effective solar–thermal cost contribution per cubic meter. This is why the cost reduction is most visible in the AD-only case, where internal recovery compensates for the lower intrinsic productivity of the base cycle. The additional reductions from EJ and HDH remain substantial even with HR, because they act on different loss mechanisms: ejectors improve vapor utilization and pressure matching, while HDH converts condenser heat into additional distillate rather than rejecting it. The combined result is a strong compounding effect: HR reduces the thermal burden, and EJ–HDH increases the water yield from the remaining heat. This synergy is reflected in the persistent separation between the three curves across all months.

Figure 9b reports the same comparison when the driving heat is supplied as waste heat, again with HR applied within the AD loop. The absolute costs are much lower than in the solar case, and the seasonal variation is reduced. The AD-HR case ranges approximately from 0.66 to 1.00 $/m^3^, with the minimum around 0.66 in June and the maximum close to 1.0 $/m^3^ in January. Adding ejector enhancement (AD-HR-EJ) reduces the cost to about 0.39–0.55 $/m^3^, with mid-year values near 0.39$/m^3^ in June and higher winter values near 0.55 $/m^3^ in January. The full AD-HR-EJ-HDH configuration remains the least expensive, typically around 0.38–0.52 $/m^3^, with the best months near 0.38 in June and winter values around 0.52 $/m^3^ in January.

These results confirm that the main economic advantage of waste heat comes from raising the water output per unit installed hardware, because the thermal energy itself is assumed to be low-cost. HR contributes by lowering the auxiliary heating demand and stabilizing the cycle operation, but the largest incremental reduction still comes from ejector and HDH integration, which increases the total distillate production without a proportional increase in capital cost. The relatively small month-to-month variation suggests that, when the energy cost is not dominant, the system economics become less sensitive to seasonal solar availability and more dependent on the performance shifts caused by ambient temperature and cooling effectiveness. For practical deployment in Saudi Arabia, this figure supports the conclusion that the tri-hybrid system is most economically attractive at industrial sites with accessible waste heat, while still offering substantial cost reductions relative to AD-only operation.

Table 4 highlights the clear performance advantage gained by employing the SP/CaCl_2_ composite within an intensified hybrid desalination architecture. In the present Riyadh study, SP/CaCl_2_ enables the AD–EJ–HDH configuration (without HR) to reach SDWP = 55 m^3^/ton·day and GOR = 2.9, which already exceeds or matches several silica-gel-based ejector/HDH hybrids while maintaining competitive costs (1.88 $/m^3^ with solar and 0.76 $/m^3^ with waste heat). The benefit becomes more pronounced when internal evaporator–condenser heat recovery is applied, where the proposed AD–HR–EJ–HDH system attains SDWP = 155 m^3^/ton·day and GOR = 3.1 with notably low freshwater costs (1.34 $/m^3^ solar and 0.38 $/m^3^ waste heat), outperforming representative benchmarks such as the silica–gel AD2EJ/HDH + HR case (83.1 m^3^/ton·day, GOR = 2.7, 1.49/0.54 $/m^3^). Compared with earlier SP/CaCl_2_ studies in Assiut, where AD-HR alone delivered lower efficiency (GOR = 0.84) and higher solar cost (3.8 $/m^3^), the current results demonstrate that the full potential of SP/CaCl_2_ is realized when it is coupled with ejector-based vapor utilization and HDH heat cascading. Overall, the comparison confirms that SP/CaCl_2_ is not only a high-capacity adsorbent, but also a strong enabler for high-output and cost-competitive hybrid adsorption desalination in hot-climate regions, positioning the present study as a meaningful step toward practical deployment in Saudi Arabia.

## 4. Conclusions

This study proposed and assessed a hybrid solar-driven desalination system tailored for Saudi Arabian conditions (Riyadh), integrating adsorption desalination (AD) using an SP/CaCl_2_ composite adsorbent with a dual-ejector stage and a humidification–dehumidification (HDH) unit. The integration was designed to intensify the energy utilization, upgrade the condenser waste heat into additional freshwater production, and improve the overall thermal performance under hot-climate operation. Two operating modes were investigated, including a production-oriented configuration that applies internal evaporator–condenser heat recovery (HR) when no cooling effect is required. The system was evaluated using a validated numerical framework under realistic monthly meteorological inputs, and the performance was quantified using the SDWP, GOR, SCP, and the freshwater cost under both solar and waste-heat driving.

Key findings of the study include the following:

– Hybridization effect (without HR): The integrated AD–EJ–HDH configuration achieved an SDWP around 41–56 m^3^/ton·day and a GOR about 2.6–2.9 over the year, with the best performance occurring in high-irradiance months. The freshwater cost ranged from 1.8 to 2.4 $/m^3^ under solar driving and 0.70 to 0.90 $/m^3^ under waste heat.
– Role of internal evaporator–condenser heat recovery (HR): When HR was applied (for freshwater production mode, no cooling demand), the system performance increased substantially to an SDWP around 95–155 m^3^/ton·day and a GOR about 2.9–3.1. The corresponding costs decreased to 1.2–2.1 $/m^3^ (solar) and 0.38–0.52 $/m^3^ (waste heat), confirming that internal heat recovery strengthens both the productivity and energy utilization.
– Cooling-related indicators (AD sub-cycle): The adsorption unit maintained seasonal SCP values of approximately 320–430 W/kg and COP values around 0.66–0.72, indicating stable adsorption-cycle behavior across the year, despite the large ambient temperature variations in Riyadh.
– Material contribution and comparative advantage: Under identical system configuration and operating conditions, SP/CaCl_2_ delivered about 85% higher freshwater production than silica gel, demonstrating that polymer–salt composites can provide a strong system-level advantage when combined with ejector intensification and HDH heat cascading.
– Subsystem contribution trends: The production-share results showed that the ejector-assisted vapor utilization represents the dominant contribution to total freshwater output, while HDH provides a consistent secondary gain by converting condenser heat into additional distillate. This distribution remained relatively stable across the year, suggesting robust hybrid operation under seasonal variability.

In conclusion, the proposed SP/CaCl_2_-based AD–EJ–HDH system provides a high-performing and cost-competitive pathway for low-grade heat desalination in Saudi Arabia. The HR-enabled production mode is particularly effective when cooling is not required, delivering the highest SDWP and the lowest unit water cost. The results support the development of hybrid adsorption-based desalination plants that exploit both solar thermal energy and industrial waste heat, and they highlight the added value of polymer–salt composite adsorbents in hot-climate regions.

Despite the promising outcomes, the long-term system reliability will depend on the adsorbent durability and stable bed performance, especially given the potential swelling and property changes of SP/CaCl_2_ under repeated thermal cycling. In addition, pilot-scale validation is needed to confirm the vacuum stability, heat-exchanger fouling behavior, and HDH packing performance with real seawater. Future work will focus on experimental implementation, the cyclic durability testing of SP/CaCl_2_ in structured beds, and the techno-economic optimization for full-scale deployment in Saudi operating conditions.

## Figures and Tables

**Table 1:** AD SP/CaCl_2_ model [11].

Component	Governing Equation *	Eq.
Sorption bed	${\left[{{\left(m\;c_{p}\right)}_{Cu+Al}+\left(m\;c_{p}\right)}_{sp}{+m}_{sg}{c_{p}}_{v}C\right]}^{bed}\frac{{dT}_{bed}}{dt}=m_{sp}H_{st}\frac{dC}{dt}-{\dot{m}}_{w}{c_{p}}_{w}{\left(T_{w,out}-T_{w,in}\right)}^{bed}$	(1)
Heat of adsorption	$H_{st}=h_{fg}+E\left(ln{\left(\frac{C_{o}}{C}\right)}^{\frac{1}{n}}\right)+\frac{E\;T\;\alpha }{n}{\left(\ln\left(\frac{C_{o}}{C}\right)\right)}^{\frac{1-n}{n}}$	(2)
Adsorption evaporator	${\left[{\left(m\;c_{p}\right)}_{Cu}+{\left(mc_{p}\right)}_{w}\right]}^{AD-ev}\;\frac{{dT}_{AD-ev}}{dt}=\theta h_{f}{\dot{m}}_{sw,in}-{\varphi .h}_{fg}{\left(\frac{dC}{dt}\right)}_{ads}m_{sp}+{\dot{m}}_{ch}{c_{p}}_{ch}\left(T_{ch,in}-T_{ch,out}\right)-{\gamma h}_{b}{\dot{m}}_{b}$	(3)
	$m_{sw,ev}\frac{{dX}_{sw,ev}}{dt}={\theta X}_{sw,in}{\dot{m}}_{sw,in}-{\gamma X}_{sw,in}{\dot{m}}_{b}-{\varphi .X}_{D}{\left(\frac{dC}{dt}\right)}_{ads}m_{sp}$	(4)
	$\frac{{dm}_{sw,ev}}{dt}=\theta {\dot{m}}_{sw,in}-\gamma {\dot{m}}_{b}-\varphi {\left(\frac{dC}{dt}\right)}_{ads}m_{sp}$	(5)
Ejector evaporator	${\left\{{\left[{\left(mc_{p}\right)}_{cu}+{\left(mc_{p}\right)}_{w}\right]}^{ev}\;\frac{{dT}_{ev}}{dt}\right\}}_{Ej-ev}={\left\{\theta h_{f}\left(T_{ev},X_{sw,ev}\right){\dot{m}}_{sw,in}-h_{fg}\left(ER_{1}+ER_{2}\left(ER_{1}+1\right)\right)\times \varphi {\left(\frac{dC}{dt}\right)}_{des}m_{sp}+{\dot{m}}_{ch}{c_{p}}_{ch}\left(T_{ch,in}-T_{ch,out}\right)-{\gamma h}_{b}\left(T_{ev},X_{s,ev}\right)m_{b}^{\cdot }\right\}}_{Ej-ev}$	(6)
	${\left[\frac{{dm}_{sw,ev}}{dt}\right]}_{Ej-ev}={\left[\theta \;{\dot{m}}_{sw,in}-\gamma {\dot{m}}_{b}-\left(ER_{1}+ER_{2}\left(ER_{1}+1\right)\right)\times \varphi {\left(\frac{dC}{dt}\right)}_{des}m_{sp}\;\right]}_{Ej-ev}$	(7)
Adsorption condenser	${\left[{\left(m\;c_{p}\right)}_{Cu+ir}+{\left(m\;c_{p}\right)}_{w}\right]}^{AD-co}\;\frac{{dT}_{AD-co}}{dt}=\varphi h_{fg}{\left(\frac{dC}{dt}\right)}_{des}m_{sp}+{\dot{m}}_{m}\;{c_{p}}_{w}{\left(T_{w,in}-T_{w,out}\right)}^{AD-co}$	(8)
Ejector condenser	${\left[{\left(m\;c_{p}\right)}_{Cu+ir}+{\left(m\;c_{p}\right)}_{w}\right]}^{Ej-co}\;\frac{{dT}_{Ej-co}}{dt}=Y.\varphi .h_{fg}\left(1+ER_{1}\right)\left(1+ER_{2}\right){\left(\frac{dC}{dt}\right)}_{des}m_{sp}+{\dot{m}}_{m}\;{c_{p}}_{w}{\left(T_{w,in}-T_{w,out}\right)}^{Ej-co}$	(9)
Outlet temperature from HXs (condensers and evaporators)	$T_{w,out}=T_{hex}+\left(T_{w,in}-T_{hex}\right)\;exp\left(\frac{-{UA}_{hex}}{{\left(\dot{m}c_{p}\right)}_{w}}\right)$	(10)
Adsorption kinetics	$C_{eq}=C_{o}\exp\left\{-{\left(\frac{RT}{E}\ln\left(\frac{P_{s}}{P}\right)\right)}^{n}\right\}$	(11)
	$\frac{dC}{dt}=\frac{F_{o}D_{s}}{R_{p}^{2}}\left(C_{eq}-C\right)$	(12)
	$D_{s}=D_{so}\;exp\left(\frac{-E_{a}}{RT}\right)$	(13)

* The symbols are defined in the nomenclature.

**Table 2:** The governing equations for the humidifier and dehumidifier [38,39].

Component	Governing Equation *	Eq.
Dehumidifier	${{\dot{m}}_{w}}_{HDH}={\dot{m}}_{a}\left(\omega_{in}-\omega_{out}\right)$	(14)
	${\dot{m}}_{a}\left(h_{15}-h_{16}\right)-{{\dot{m}}_{w}}_{HDH}\;h_{13}={\dot{m}}_{sw}\left(h_{18}-h_{17}\right)$	(15)
	$\varepsilon_{deh}=\max\left(\frac{h_{18}-h_{17}}{h_{18,ideal}-h_{17}},\frac{h_{15}-h_{16}}{h_{15}-h_{16,ideal}}\;\right)$	(16)
Humidifier	${{\dot{m}}_{w}}_{HDH}={\dot{m}}_{sw}-{\dot{m}}_{b,HDH}={\dot{m}}_{a}\left(\omega_{out}-\omega_{in}\right)$	(17)
	${\dot{m}}_{a}\left(h_{15}-h_{16}\right)+{\dot{m}}_{b,HDH}\;h_{14}={\dot{m}}_{sw}h_{8}$	(18)
	$\varepsilon_{hum}=\max\left(\frac{h_{15}-h_{16}}{h_{15,ideal}-h_{16}},\frac{h_{8}-h_{14}}{h_{8}-h_{14,ideal}}\;\right)$	(19)

* The symbols are defined in the nomenclature.

**Table 3:** The capital cost of the proposed system.

ADHDH Unit		
Solar collector	300×No. of solar collectors	
Adsorption beds	240	[42]
Adsorbent	68	
Humidifier and dehumidifier	203	
Evaporator	150	
Condenser	120	
Water tanks	250	
Pumps and blowers	345	
Accessories/fittings/pipes	220	
Ejectors	2×200	

**Table 4:** Benchmarking of the proposed SP/CaCl_2_-based AD–EJ–HDH system against representative adsorption-driven hybrid desalination previous studies.

System Configuration	Adsorbent Pair	SDWP (m^3^/ton·day)	GOR	SCP (W/kg)	Freshwater Cost (Solar) ($/m^3^)	Freshwater Cost (Waste Heat) ($/m^3^)
AD–EJ–HDH (without HR) Current study (Riyadh)	SP/CaCl_2_	55	2.9	430	1.88	0.76
AD–HR–EJ–HDH Current study (Riyadh)	SP/CaCl_2_	155	3.1	-	1.34	0.38
AD2EJ/HDH + HR (Assiut) [11]	Silica gel	83.1	2.7	-	1.49	0.54
AD + 2 ejectors + internal HR (AD2EJ-HR)[11]	Silica gel	67	2.22	—	1.74	0.57
AD + 2 ejectors (AD2EJ) without HR [11,43]	Silica gel	22.9	1.61	220	2.94	1.33
AD-HR, Assiut, Egypt) [44]	SP/CaCl_2_	45	0.84	425	3.8	0.63
AD-HR-HDH, Assiut, Egypt) [45]	SP/CaCl_2_	77.3	1.87	-	1.83	0.49
AD–HR-HDH-(Assiut, Egypt) [32]	Silica gel	24.2	1.35	-	3.1	1.2
AD–HR-HDH Assiut, Egypt) [32]	Silica gel/CaCl_2_	59	1.8	-	2.0	0.59

## Data Availability

All data generated or analyzed during this study are included in this published article and its Supplementary Information Files.

## Supplementary Materials

The following supporting information can be downloaded at https://www.mdpi.com/article/10.3390/polym18040450/s1, Figure S1: SP/CaCl2 composite preparation method; Figure S2: The XRD analysis; Figure S3: ADS modeling flow chart; Figure S4: A comparison of GOR predicted by the developed HDH simulation model at different mass ratios (MR) with the corresponding previous; Table S1: SP porous properties; Table S2: D-A isotherm models values; Table S3: Numerical parameters and operation conditions; Table S4: SDWP and COP of the AD system obtained from the present model and previous experimental measurements; Table S5:Validation of the model of the L-V ejector at condenser temperature of 40 °C; Table S6: Deviation between the present results and data given in Ref for V-V ejector; Table S7: Error analysis; Table S8: Sensitivity analysis of design parameters on adsorption desalination outcomes; Table S9: Sensitivity analysis for hybrid system outcomes. References [8,11,17,46,47,48,49,50,51,52,53,54,55] are cited in Supplementary Materials.

## Nomenclature

Symbols	
A	Area, m^2^
C	Adsorption uptake, kg/kg
C_o_	Maximum adsorption uptake, kg/kg
c_p_	Specific heat, J/kg·K
D_s_	Surface diffusivity, m^2^/s
D_so_	Constant
E	Characteristic energy, J/mol
E_a_	Activation energy, J/mol
ER	Entrainment ratio
F_o_	Constant
h	Specific enthalpy, J/kg
h_fg_	Latent heat of vaporization, J/kg
H_st_	Heat of adsorption, J/mol
i	Interest rate
m	Mass, kg
$\dot{m}$	Mass flow rate, kg/s
n	Isotherm constant
P	Pressure, Pa
$\dot{Q}$	Heat, W
Ṝ	Universal gas constant, J/mol·K
Rp	Particle radius of adsorbent, m
s	Specific entropy, J/kg·K
${\dot{S}}_{gen}$	Entropy generation, W/K
t	Time, s
T	Temperature, °C
UA	Overall heat transfer coefficient, W/K
v	Velocity, m/s
$\dot{W}$	Power, W
X	Seawater salinity, ppm
Y	Number of years
Z	Cost, $
$\dot{Z}$	Annual cost, $/yr
Greek	
α	Thermal expansion, 1/K
µ	Chemical potential, J/mol
η	Efficiency, %
ν	kinematic viscosity, m^2^/s
τ	Number of cycles per day, 1/day
ε	Effectiveness
γ, φ, θ	Constants equal 0 or 1
ω	Humidity ratio, kg/kg
Subscripts	
1, 2, …	State
amb	Ambient
ads	Adsorption
Al	Aluminum
ch	Chilled water
co	Condenser
Cu	Copper
cw	Cooling water
des	Desorption
deh	Dehumidifier
dr	Driving
Ej	Ejector
eq	Equilibrium
ev	Evaporator
hex	Heat exchanger
hum	Humidifier
hw	Hot water
ideal	Ideal conditions
in	Inlet
ir	Iron
l	Labor
L-V	Liquid–vapor
m/o	Maintenance/operation
out	Outlet
sw	Seawater
T	Total
v	Vapor
V-V	Vapor–vapor
w	Water
Superscripts	
AD-co	Adsorbed bed—condenser
AD-ev	Adsorbed bed—evaporator
Ej-co	Ejector—condenser
Abbreviations	
AD	Adsorption desalination
EJ	Ejector
F	Capital recovery factor
GOR	Gained output ratio
HDH	Humidification–dehumidification
HR	Heat recovery
SCP	Specific cooling power, W/kg
SDWP	Specific daily water production, m^3^/ton/day
SEC	Specific energy consumption, kWh/m^3^
