# Supplementary material for: High-Productivity Hybrid Adsorption Desalination Using a Sodium Polyacrylate/CaCl2 Composite with Dual Ejectors and Humidification–Dehumidification Under Saudi Arabian Climate Conditions
Source: Polymers (Basel). 2026 Feb 10;18(4):450. doi: 10.3390/polym18040450 (PMC12944319; doi:10.3390/polym18040450)
Supplement: Supplementary file 1 [file polymers-18-00450-s001.zip › polymers-4094855-supplementary.pdf]

SUPPLEMENTARY

High-Productivity Hybrid Adsorption Desalination Using a Sodium Polyacrylate/$CaCl_2$ Composite with Dual Ejectors and Humidification–Dehumidification under Saudi Arabian Climate Conditions

Ridha Ben Mansour[1], Ahmed S. Alsaman[1], Ehab S. Ali[1,*], Ahmed E. Abu El-Maaty[2], Rached Ben-Mansour[1,2]

[1]Interdisciplinary Research Center for Sustainable Energy Systems, KFUPM, Dhahran, 31261, Saudi Arabia
[2]Mechanical Engineering Department, KFUPM, Dhahran 31261, Saudi Arabia

* Corresponding Author. Email: ehab.abdelaal@kfupm.edu.sa

1. Adsorbent Materials

1.1 Material

Sodium polyacrylate (SP) is the sodium salt of poly (acrylic acid) and behaves as an anionic polyelectrolyte due to the negatively charged carboxylate groups along its backbone. The presence of sodium ions promotes strong hydration, allowing SP to absorb very large quantities of water, reported to reach nearly 300. SP also exhibits promising practical characteristics, including mechanical stability, high hydration capacity, and thermal resistance [1,2] . It was selected in this work because it can retain water effectively within its porous polymer structure and is considered non-toxic. A key limitation of SP is its pronounced swelling behavior, which can influence the tracking density and control-volume design. The SP used in this study was supplied as white granules with a particle size in the range of 0.075–0.8 mm and a density of about 740 kg.m^{-3}.
Because swelling may impose constraints on the adsorption-bed geometry and operation, the swelling behavior of SP was examined before its use in adsorption applications. Figure S1 compares the swelling of SP and activated SP-HCl after 30 days. As expected, raw SP undergoes substantial swelling. In contrast, acid-treated samples exhibit a progressive reduction in swelling as the HCl

concentration increases from 2 to 8 M, indicating that acid treatment can enhance dimensional stability and potentially facilitate the use of SP-based materials in compact control-volume and bed configurations.

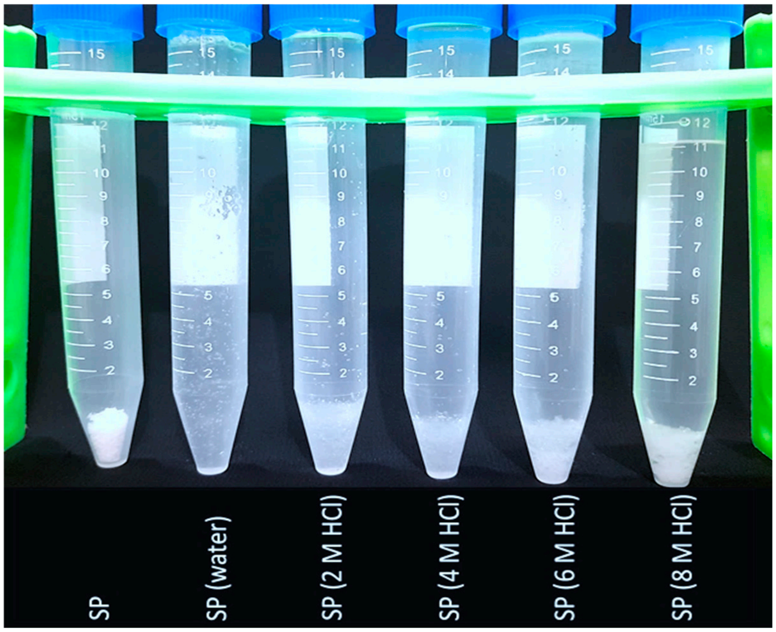

Figure S1. SP Swelling at different HCl activation after 30 days [1].

1.2 Pretreatment and preparation

Acid activation using HCl has been widely applied for adsorbent pretreatment and has been reported in the literature [3–6]. Based on prior optimization, an HCl concentration of 2 M was identified as effective for improving surface characteristics and adsorption capacity. Accordingly, SP was pretreated with 2 M HCl in this work. In addition, modifications were investigated: SP with SP/$CaCl_2$ composite prepared by direct impregnation. For the composite, 2.1 g of dried SP was impregnated with a 30 wt.% loading of $CaCl_2$ hydrate dissolved in distilled water.

1.3 X-ray diffraction

The structural features of raw SP, SP/HCl, and SP/$CaCl_2$ were analyzed using X-ray diffraction (XRD) with a Bruker Axs-D Advance diffractometer employing Cu-Kα radiation (λ = 1.54060 Å)

over a 2θ scan range of 20–60°. The experimental procedure follows the methodology reported in Refs. [7,8]. The XRD patterns (Figure S3) reveal multiple reflections, indicating the presence of crystalline components, including NaCl-related phases (CN) and halite (CNH). Raw SP exhibits a CN peak near 32°, while CNH reflections around 45.6° appear in the treated SP/HCl and SP/$CaCl_2$ samples. Notably, SP/$CaCl_2$ shows the fewest and weakest peaks, indicating a more amorphous structure and suggesting a lower level of crystalline impurities compared with the other samples.

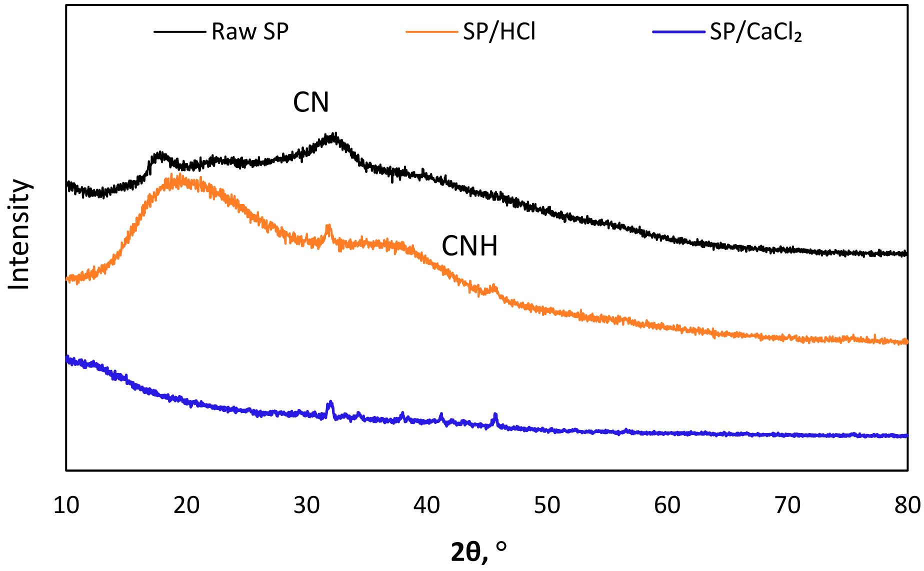

Figure S2. The XRD analysis [1].

1.4 Nitrogen adsorption isotherm

The properties and porosity of SP samples were evaluated using N_2 adsorption–desorption isotherms measured at −196 °C with a Quantachrome TouchWin™ system. Prior to measurement, samples were degassed for 10 h at 150 °C. The Brunauer–Emmett–Teller (BET) surface area (S_BET) was calculated from adsorption data at P/P_0 = 0.3, while the total pore volume was estimated at P/P_0 = 0.99 [1].

As summarized in Table S1, the obtained N_2 isotherms correspond to a Type III behavior, and the pore-size distribution falls within the mesoporous range (2–50 nm) according to IUPAC classification [1].

Table S1. SP porous properties[1].

Adsorbent	S_{BET} (m^2/g)	$V_{0.99}$ (cm^3/g)	Average pore size (nm)	R_p (nm)
**Raw SP**	105.3	0.168	3.188	12.91
**SP/HCl**	97.46	0.1554	3.188	13.992
**SP/$CaCl_2$**	103.953	0.1657	3.188	13.118

1.5 Water vapor adsorption

The extracted D–A parameters and the associated thermodynamic quantities are listed in Table 3. Based on the experimental measurements and fitted results, the average isosteric heat of adsorption (Hst) was also evaluated and is reported in Table 3 to support comparison of the adsorption energetics among the studied SP-based materials.

Table S2. D-A isotherm models values [1].

Adsorbent	C_O (kg/kg)	E (kJ/kg)	n (-)	Average H_{st} (kJ/kg)
**Raw SP**	1.5	700.6	0.744	2600
**SP/HCl**	1	932.6	0.54	2668
**SP/$CaCl_2$**	1.33	2087.3	1.522	2633

2. Modelling parameters and Flowcharts

Table S3. Numerical parameters and operation conditions [9,10].

Parameter	value	Unit	Parameter	value	Unit
UA_{con}	300	W/K	Cp_{ir}	450	J/kg.K
UA_{bed}	500		Cp_w	4180	
UA_{eva}	300		Cp_v	4190	
M_{bed}	15	kg	R	8314	J/mol.K

M_{con}	7.5		i	5%	-
M_{eva}	7		Y	30	Year
$M_{w,eva}$	3		F	0.9	-
M_{ac}	1.8		W	8.11%	-
Cp_{cu}	386	J/kg.K	β	4.63%	-
$\dot{m}_{hw}$	0.20	kg/s	$\dot{m}_{ch}$	0.20	kg/s
$\dot{m}_{cw}$	0.30	kg/s	T_h	50-95	°C
T_c	25	°C	$T_{chill,in}$	25	°C

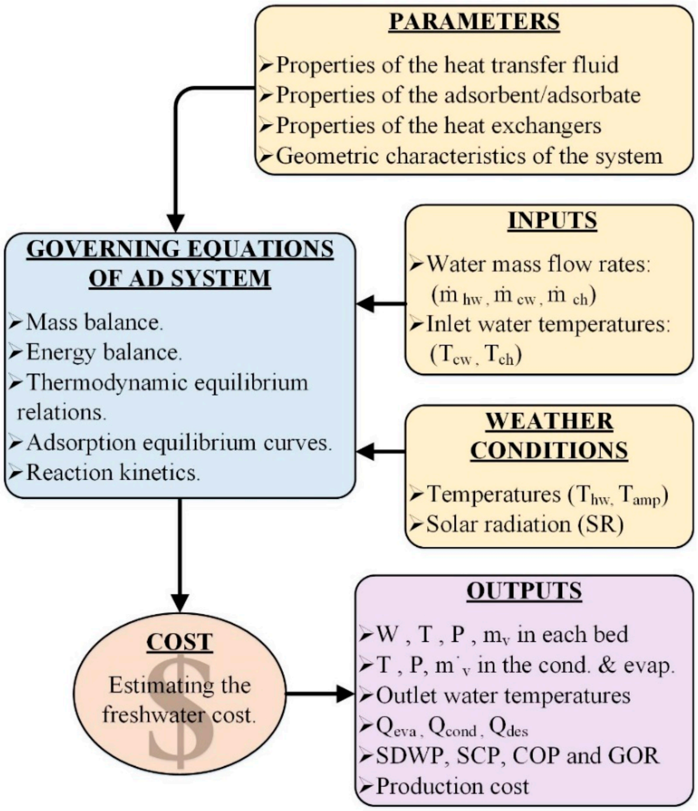

Figure S3. ADS modeling flow chart.

3. Model Validation

The current model is validated in three stages: assessing the AD model, the liquid–vapor (L-V) model, and the vapor-vapor (V–V) model. For each step, the outcomes are compared with the data available in the existing literature.

3.1 Validation of the AD model

The present AD numerical model is validated using the experimental measurements reported in Ref. [11] under the same operating conditions.

To verify the accuracy of the AD numerical model, results were compared with experimental data published in Ref. [11], using identical operating conditions. A summary of both the simulated and measured results for the ADS system is presented in Table S4. The data show that the numerical model closely matches the experimental findings. Specifically, the maximum deviation between the simulation and experiment is less than 5% for SDWP and under 4% for COP. These small margins of error indicate a strong correlation between the model and actual measurements, confirming that the numerical model is reliable for analyzing the performance of the AD cycle.

Table S4. SDWP and COP of the AD system obtained from the present model and previous experimental measurements [11].

**Cycle No.**	**SDWP (m^3/ton/day)**			**COP (–)**		
	Exp. data	Present data	Error (%)	Exp. data	Present data	Error (%)
1	4.15	4.2	1.20	0.44	0.451	2.50
2	4.01	4.13	2.99	0.448	0.46	2.68
3	3.95	4.07	3.04	0.454	0.472	3.96
4	3.82	4.0	4.71	0.46	0.475	3.26
5	3.8	3.9	2.63	0.453	0.462	1.99
6	3.7	3.8	2.70	0.45	0.457	1.56

3.2 Validation of the liquid to vapor ejector

To validate the liquid-to-vapor (L-V) ejector model, results from the current simulation were compared with those published in Ref. [12] , using R134a as the working fluid. Table S5 shows

the relative error between the present model and the literature values. The average deviation in the outlet pressure of the L-V ejector is found to be less than 0.7%, and the maximum error in the entrainment ratio is just 0.127%. These minimal differences confirm that the model accurately captures the performance of the L-V ejector.

Table S5. Validation of the model of the L-V ejector at condenser temperature of 40 ºC.

T_{ev} (ºC)	ER (–)			$P_{EJ\text{-}cond}$ (kPa)		
	Previous data [12]	Present data	Error (%)	Previous data [12]	Present data	Error (%)
10	0.789	0.790	0.127	437.1	435	0.480
5	0.762	0.7625	0.066	375.0	376.25	0.333
0	0.7359	0.7368	0.122	320.4	321.8	0.437
-5	0.712	0.712	0.000	272.4	273.8	0.514
-10	0.6879	0.6873	0.087	230.5	232.0	0.651

3.3 Validation of vapor to vapor ejector

For the vapor-to-vapor (V-V) ejector, the model was validated against the results reported in Ref. [13], with water as the working fluid. Under identical operating conditions, the variation between the model's predictions and the reference data is under 3.0%, as illustrated in Table S6. This close agreement confirms the validity of the V-V ejector model and its compatibility with the AD system for evaluating the overall performance of the proposed setup.

Table S6. Deviation between the present results and data given in Ref. [13] for V-V ejector.

T_{ev} (ºC)	T_{co} (ºC)	ER (Reported)	ER (present)	Error %
**15**	80	0.265	0.26	1.887
**15**	85	0.293	0.29	1.024
**15**	90	0.318	0.325	2.201
**15**	95	0.352	0.36	2.273
**15**	100	0.379	0.39	2.902
**15**	105	0.41	0.42	2.439
**15**	110	0.438	0.447	2.055
**10**	100	0.325	0.318	2.154
**10.8**	100	0.356	0.347	2.528
**11.7**	100	0.39	0.395	1.282
**12.5**	100	0.428	0.434	1.402
**13.3**	100	0.47	0.475	1.064
**14.17**	100	0.515	0.502	2.524
**15**	100	0.564	0.571	1.241

In addition to validating each model, entropy generation in every component was calculated and found to be positive, ensuring that the system complies with both the first and second laws of thermodynamics.

3.4 Validation of the HDH model

Finally, the HDH simulation model developed in this study was validated by benchmarking its predictions against the results reported by Sharqawy et al. [14]. As illustrated in Figure A.3, the present model reproduces the published trends with very good agreement. The remaining deviation is minor, with the maximum discrepancy between the current outputs and the reference data not exceeding **2.9%**.

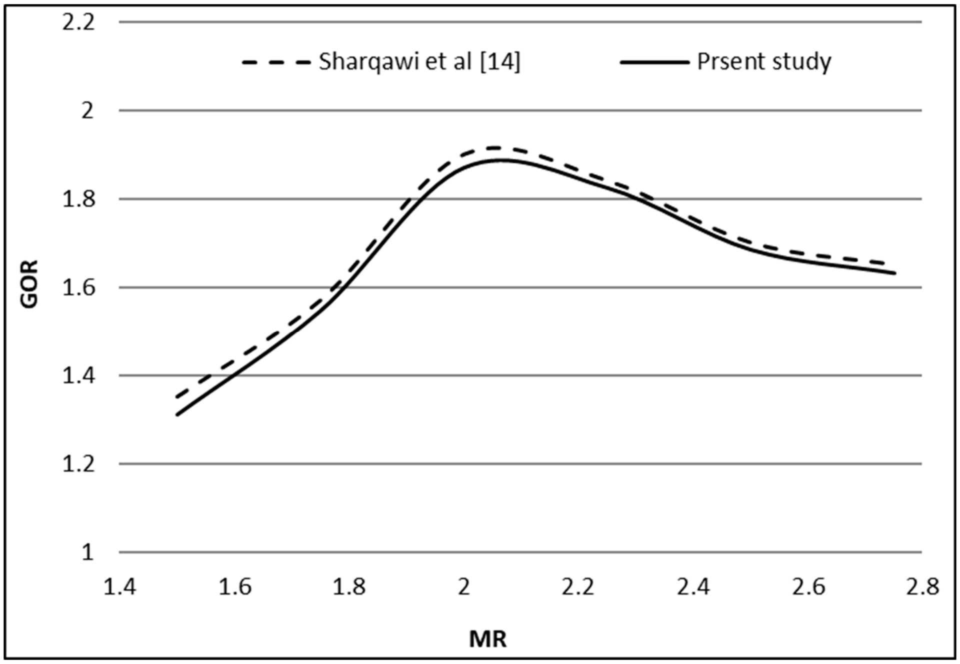

Figure S4. A comparison of GOR predicted by the developed HDH simulation model at different mass ratios (MR) with the corresponding previous.

3.5 Error analysis

Error analysis is a critical component of system evaluation, providing insights into the accuracy and reliability of the results of the conducted models. To evaluate the performance validation of

the conducted sub-models of the AD-EJ-HDH system, including AD, EJ cycle, and HDH cycle, two distinct performance metrics are employed: root mean square error (RMSE) and coefficient of determination (R^2). A model with higher accuracy is indicated by lower values for RMSE, while higher values for R^2. The formulas for these statistical metrics are presented below:

$$R^2 = \frac{\left(\sum_{i=1}^{n_s}(d_i-\bar{d})(y_i-\bar{y})\right)^2}{\sum_{i=1}^{n_s}(d_i-\bar{d})^2 \times \sum_{i=1}^{n_s}(y_i-\bar{y})^2} \quad \text{(S1)}$$

$$RMSE = \sqrt{\frac{1}{n_s}\sum_{i=1}^{n_s}(d_i - y_i)^2} \quad \text{(S2)}$$

Table S7. Error analysis results confirm the performance validation of the predictive models of ADS cycle, HDH cycle, LVEJ, and VVEJ.

Table S7. Error analysis.

Model	Output parameters	R2	RMSE
**ADS cycle**	Gained output ratio (GOR)	0.9799	0.0120
	Water productivity (L/hr)	0.9654	0.122
**VVEJ**	Entrainment ratio (-)	0.9921	0.0071
**LVEJ**	Entrainment ratio (-)	0.9999	0.0035
**HDH**	GOR	0.9782	0.0756

The developed AD, HDH, and EJ models demonstrate high prediction accuracy when validated against published experimental data, respectively. The RMSE values remained low across both performance and thermal parameters, and the coefficient of determination (R^2) exceeded 0.965 in most cases, confirming the strong agreement between simulated and reference values. This supports the robustness and reliability of the modeling framework used in this study.

3.6 Sensitivity analysis

Table S8 presents the sensitivity analysis of the adsorption desalination fresh water cost with cost parameters (capital and operations cost). The table shows that the total freshwater cost system is less sensitive to operation costs. The capital cost sensitivity reaches ±9.6% and ±7.9 % when the system is driven by solar energy and waste heat, respectively. The operation cost sensitivity reaches ±2.9 % and ±1.5 % when the system is driven by solar energy and waste heat, respectively.

Table S8. Sensitivity analysis of design parameters on adsorption desalination outcomes.

Parameters	Freshwater Cost (Powered by waste heat)	Freshwater Cost (Powered by solar energy)
Capital cost ± 10 %	±7.9 %	±9.6%
Electrical pumping cost ± 10 %	±2 %	±0.4 %
Labor cost ± 10 %	±0.6 %	±0.7 %
Maintenance cost ± 10 %	±0.31 %	±0.39 %
Operation cost ± 10 %	±1.5 %	±2.9 %

Table S9. Sensitivity analysis for hybrid system outcomes.

**Adsorbent**	**Hybrid system SDWP**	**Hybrid system SCP**	**Hybrid system GOR**
cycle time ± 10 %	±1.37 %	±1.38 %	±1.41 %
Ejector ER ± 10 %	**5.5%**	0%	5.5%
HDH desalinate water ± 10%	**1.7%**	0%	**1.7%**

References

[1] A.S. Alsaman, E.M.M. Ibrahim, M. Salem Ahmed, E.S. Ali, A.M. Farid, A.A. Askalany, Experimental investigation of sodium polyacrylate-based innovative adsorbent material for higher desalination and cooling effects, Energy Convers. Manag. 266 (2022) 115818. https://doi.org/10.1016/j.enconman.2022.115818.

[2] E.S. Ali, A.S. Alsaman, M.H.M. Tawfik, A.A. Askalany, W.M. El-Maghlany, A.E. Zohir, A.M. Farid, M. Ghazy, Solar-powered hybrid adsorption desalination/humidification-dehumidification system, Thermal Science and Engineering Progress 51 (2024) 102598. https://doi.org/10.1016/J.TSEP.2024.102598.

[3] E.S. Ali, A.A. Askalany, K. Harby, M.R. Diab, B.R.M. Hussein, A.S. Alsaman, Experimental adsorption water desalination system utilizing activated clay for low grade heat source applications, J. Energy Storage 43 (2021) 103219. https://doi.org/10.1016/j.est.2021.103219.

[4] A. Eze, J.O. Nwadiogbu, E.T. Nwankwere, A. Appl, S. Res, Effect of Acid Treatments on the Physicochemical Properties of Kaolin Clay, Arch. of Appl. Sci. Res. 4 (2012) 792–794.

[5] B.K. Aziz, M.A. Abdullah, K.J. Jubrael, Acid activation and bleaching capacity of some clays for decolourizing used oils, Asian Journal of Chemistry 23 (2011) 2449–2455.

[6] J. Temuujin, T. Jadambaa, G. Burmaa, S. Erdenechimeg, J. Amarsanaa, K.J.D. MacKenzie, Characterisation of acid activated montmorillonite clay from Tuulant (Mongolia), Ceram. Int. 30 (2004) 251–255. https://doi.org/10.1016/S0272-8842(03)00096-8.

[7] S. Nasrazadani, S. Hassani, Modern analytical techniques in failure analysis of aerospace, chemical, and oil and gas industries, in: Handbook of Materials Failure Analysis with Case Studies from the Oil and Gas Industry, Elsevier, 2016: pp. 39–54. https://doi.org/10.1016/B978-0-08-100117-2.00010-8.

[8] W.S. Mohamed, A.M. Abu-Dief, Impact of rare earth europium (RE-Eu3+) ions substitution on microstructural, optical and magnetic properties of CoFe2−xEuxO4 nanosystems, Ceram. Int. 46 (2020) 16196–16209. https://doi.org/10.1016/j.ceramint.2020.03.175.

[9] M. Ghazy, A.A. Askalany, E.M.M. Ibrahim, A.S.A. Mohamed, E.S. Ali, R. AL-Dadah, Solar powered adsorption desalination system employing CPO-27(Ni), J. Energy Storage 53 (2022) 105174. https://doi.org/10.1016/j.est.2022.105174.

[10] E.S. Ali, R.H. Mohammed, N.A.A. Qasem, S.M. Zubair, A. Askalany, Solar-powered ejector-based adsorption desalination system integrated with a humidification-dehumidification system, Energy Convers. Manag. 238 (2021) 114113. https://doi.org/10.1016/j.enconman.2021.114113.

[11] A.S. Alsaman, A.A. Askalany, K. Harby, M.S. Ahmed, Performance evaluation of a solar-driven adsorption desalination-cooling system, Energy 128 (2017) 196–207.

[12] M. Hassanain, E. Elgendy, M. Fatouh, Ejector expansion refrigeration system: Ejector design and performance evaluation, International Journal of Refrigeration (2015). https://doi.org/10.1016/j.ijrefrig.2015.05.018.

[13] B.M. Ziapour, A. Abbasy, First and second laws analysis of the heat pipe/ejector refrigeration cycle, Energy (2010). https://doi.org/10.1016/j.energy.2010.04.016.

[14] M.H. Sharqawy, M.A. Antar, S.M. Zubair, A.M. Elbashir, Optimum thermal design of humidification dehumidification desalination systems, Desalination 349 (2014) 10–21. https://doi.org/10.1016/j.desal.2014.06.016.
